# Advances and Future Challenges of Minimally Invasive Surgery in Children

**DOI:** 10.3390/children9121959

**Published:** 2022-12-13

**Authors:** Zenon Pogorelić

**Affiliations:** 1 Department of Pediatric Surgery, University Hospital of Split, 21 000 Split, Croatia; zpogorelic@gmail.com; Tel.: +385-21-556-654; 2 Department of Surgery, School of Medicine, University of Split, 21 000 Split, Croatia

Minimally invasive surgery is a relatively new field of surgery where the surgeons operate through small incisions using a variety of techniques to perform less damage to the patient’s body than with conventional open surgery [1,2,3]. The benefits of minimally invasive surgery are well-known and have been reported multiple times in medical literature. The most important benefits include: faster recovery and return to everyday activities, less pain, a shorter hospital stay, fewer complications and better cosmetic effects [1,2,3,4,5,6,7].

The goal of minimally invasive surgery is to perform operations through very small incisions with equal or superior clinical outcomes and less impact on a patient’s body and organs. Minimally invasive surgery has become very popular over the last 30 years. Minimally invasive surgery in the pediatric population was slow to advance, but due to the development of technologies and instruments adapted to newborns and small children over the last 20 years has rapidly expanded to include all major pediatric surgical procedures in infants and children [3,4,7,8,9,10]. The benefits to the patient are great, but the technical hurdles are many, because of the varied size and physiology of this patient population.

Equipment and instruments used for minimally invasive procedures in the pediatric population are specially designed to allow safe access to the small abdominal or thoracic cavities, maintain an adequate working space and perform maneuvers with the same or even better safety and efficacy as in open procedures [4,7,8,10]. The diameter of instruments and telescopes varies from 2 to 10 mm depending on the procedure and child’s age [3,4,7,10]. 

Developed 5 mm clip applicators and staplers and also advanced energy source devices, such as the harmonic scalpel, have proved to be imminently helpful in minimally invasive pediatric surgery. The equipment used for pediatric minimally invasive surgery is expensive and significantly increases the cost of treatment [4]. Recent research clearly showed that the most expensive tools used for pediatric minimally invasive surgical procedures, such as the harmonic scalpel, may be safely reused without any consequences to the patient.

More recently, the introduction of new-generation videoscopic technologies and cameras allowed three-dimensional procedures together with a high-definition video format vision in small and challenging spaces has significantly improved and therefore augmented the safety of the procedures [4,10].

A single incision laparoscopic surgery has for many pediatric surgeons become the treatment of choice in many different pediatric minimally invasive procedures. Conversion to classical three port laparoscopic or thoracic surgery can be easily performed when procedures were started through a single incision [8]. With the availability of robotic surgery in the early 2000s, some pediatric centers established robotic pediatric surgery programs.

Concerning the pediatric minimally invasive approach, in the beginning, simpler procedures, such as appendectomy, cholecystectomy, orchiopexy or hernia repair, were performed with this approach, but with the development of instruments and the advancement of technology, as well as with the acquisition of experience, pediatric surgeons began to perform laparoscopic pyloromyotomy, splenectomy, videothoracoscopy for pneumothorax or empyema or different urological procedures [4,5,7,8,9]. Finally, more and more complex surgical procedures, such as a congenital diaphragmatic hernia, repair of esophageal atresia, the Nuss repair of pectus excavatum, laparoscopic choledochal cyst excision or the minimally invasive Kasai procedure were reported [3,10]. With the availability of robotic surgery in the early 2000s, some centers established robotic pediatric surgery programs. 

The goal of this Special Issue is to present the current state of minimally invasive surgery in pediatric population and specifically focus on the trends and developments that may be expected in the coming years.
